# From awareness to action: evolving endocrinologist practices in obesity treatment in Italy

**DOI:** 10.3389/fendo.2025.1705670

**Published:** 2025-10-31

**Authors:** Marco Chianelli, Luca Busetto, Roberto Attanasio, Rinaldo Guglielmi, Renato Cozzi, Franco Grimaldi, Andrea Frasoldati, Agnese Persichetti, Enrico Papini, Antonio Nicolucci

**Affiliations:** ^1^ Unit of Endocrinology and Metabolism, Regina Apostolorum Hospital, Rome, Italy; ^2^ Department of Medicine, University of Padua, Padua, Italy; ^3^ Scientific Committee, Associazione Medici Endocrinologi, Milan, Italy; ^4^ Department of Endocrinology, Grande Ospedale Metropolitano (GOM) Niguarda Milan, Milan, Italy; ^5^ Endocrinology Unit, University Hospital S.M. Misericordia, Udine, Italy; ^6^ Endocrinology Unit, Azienda USL-IRCCS di Reggio Emilia, Reggio Emilia, Italy; ^7^ Ministry of Interior - Department of Firefighters, Public Rescue and Civil Defense, Rome, Italy; ^8^ Center for Outcomes Research and Clinical Epidemiology – CORESEARCH Srl, Pescara, Italy

**Keywords:** early treatment, educational activities, pharmacological treatment, liraglutide, obesity survey

## Abstract

**Introduction:**

Early treatment of obesity is essential and requires proactive engagement from healthcare providers. A 2022 survey among the members of the Italian Association of Clinical Endocrinologists (AME) revealed that they often did not address or manage the condition. This study aims to examine if attitudes and practices of Italian endocrinologists in managing obesity have changed in recent years, following several educational initiatives promoted by AME and the upcoming availability of specific treatments.

**Methods:**

This research utilized a web-based survey distributed to members of AME. The survey explored endocrinologists’ approaches to obesity management, covering treatment methods, referral practices, and perceived challenges in providing effective care. At the time of the survey semaglutide and tirzepatide were not commercially available in Italy for the treatment of obesity/overweight.

**Results:**

The survey received responses from 16.6% of AME members (424 out of 2560). Among respondents, 32.5% 37 identified obesity and nutrition as key areas of focus. Among participants, 43.4% reported that over 35% of the patients they see each month have obesity, compared to 37.8% in the previous survey. The proportion of endocrinologists who reported managing obesity themselves, either independently or as part of a dedicated team, slightly increased from 42.6% 40 to 45.5%. In terms of pharmacological treatment, Liraglutide was prescribed to 25% of suitable patients (IQR 5-50), as compared to 10% (IQR 0-30) in the previous survey. Participants reported with less worries about side effects of treatment and patient resistance. Poor effectiveness almost disappeared among the reported obstacles to prescribing anti-obesity drugs. High treatment costs were cited as a primary barrier to prescribing anti-obesity medications, significantly affecting long-term adherence.

**Discussion:**

Addressing the unmet educational needs of endocrinologists is essential to enhance their awareness of obesity and increase their confidence in managing this widespread condition, which has a profound impact on individuals and society.

## Introduction

The increasing prevalence of overweight and obesity has become a global health crisis, raising serious concerns worldwide. In 2021, an estimated 1.00 billion adult males and 1.11 billion adult females had overweight or obesity (1). From 1990 to 2021, the global deaths and disability adjusted life years (DALYs) attributable to high body mass index (BMI) increased more than 2.5-fold for females and males (2), with cardiovascular disease and the composite of diabetes and kidney diseases representing the two primary contributors to high BMI-attributable DALYs.

Recent estimates from the World Health Organization (WHO) indicate that overweight and obesity affect almost 60% of adults and nearly one in three children (29% of boys and 27% of girls) in the WHO European Region (3). In Italy, data from the National Institute of Statistics show that between 2001 and 2010, the number of individuals with overweight increased by approximately two million, while those with obesity rose by over one million. By 2023, over 23 million Italian adults, accounting for 46.3% of the population, were classified as overweight or obese (17.0 million with overweight and 5.8 million with obesity) (4).

Excess weight imposes a significant clinical, social, and economic burden due to its association with numerous health conditions, including diabetes, cardiovascular diseases, respiratory disorders, certain cancers, and osteoarticular diseases (5). Additionally, individuals with obesity often face stigma, discrimination, and a decline in both mental well-being and health-related quality of life (6).

Given these challenges, obesity should be acknowledged by healthcare providers and insurers as a chronic condition requiring care, support, and follow-up (7).

Despite the existence of evidence-based guidelines, including the recent Italian guidelines (8–14), the rising prevalence of obesity indicates that these recommendations are not effectively implemented. Suboptimal care for individuals with obesity is widely reported (15–18), with low rates of diagnosis, documentation, and management (19, 20), as well as insufficient knowledge of obesity treatment guidelines among healthcare professionals (21). Additionally, few individuals with obesity receive weight-loss counseling, and only about a quarter of those counseled have a follow-up appointment scheduled to monitor their progress (19, 21).

Research also highlights significant variability in the use of pharmacological and surgical interventions (19, 22), suggesting a lack of familiarity with treatment initiation and referral criteria. Misconceptions regarding the safety and effectiveness of available weight-loss medications and bariatric procedures further contribute to the inconsistent management of obesity (17, 23). Clinical endocrinologists play a crucial role in identifying, evaluating, and managing obesity, given the high proportion of patients with excess body weight in their care. A previous survey conducted by the Italian Association of Clinical Endocrinologists (AME) in 2022 documented that endocrinologists regularly encounter patients with obesity, yet they often do not address the problem or manage it (24). Furthermore, the survey showed that, despite the current availability of safe and effective anti-obesity medications, with even more efficacious options emerging, pharmacological treatment was rarely prescribed by most participants, who perceived several barriers to effective obesity management. Following the survey, AME launched a series of educational activities for its members focused on the management and care of obesity by endocrinologists.

The current study aimed to assess whether these educational activities, along with increasing body of evidence supporting the safety and effectiveness of new anti-obesity drugs, particularly glucagon-like peptide 1 (GLP1) receptor agonists, had an impact on practices and attitudes of Italian endocrinologists regarding care of people with obesity. At the time of the survey, semaglutide and tirzepatide had not yet been approved for commercial use in Italy for the treatment of obesity or overweight with complications (BMI >27).

## Materials and methods

This study was a cross-sectional survey conducted among endocrinologists who are members of AME. Participants were invited to complete an online multiple-choice questionnaire, which remained open for four weeks. The survey was created using LimeSurvey, an open-access platform offering various question templates.

A survey link was emailed to 2560 endocrinologists, with weekly reminders sent to non-respondents to encourage participation. Participation was voluntary, with no compensation provided, and all responses were anonymous. The survey data was collected and securely stored electronically by the survey platform, accessible only via a password-protected system. The survey system automatically prevented multiple submissions from the same IP address.

The questionnaire aimed to evaluate several aspects of endocrinologists’ approach to obesity management, including:

1. The frequency with which obesity (BMI ≥30 kg/m²) is addressed in clinical practice.
2. Methods used to manage obesity.
3. The initial treatment approach based on different obesity severity levels.
4. Beliefs about the ketogenic diet and meal replacements.
5. Types of physical activity recommended for individuals with obesity.
6. Attitudes toward recommending psychological support as part of obesity management.
7. Frequency of anti-obesity medication use and factors influencing drug selection.
8. Key barriers to prescribing obesity medications.
9. Major factors affecting patient adherence to treatment.
10. Awareness of emerging anti-obesity medications and expectations regarding their benefits.

The questionnaire also collected demographic information about participants, including their sex, age, years of practice, primary areas of specialization, type and setting of their practice, and whether their facility had a dedicated obesity specialist or unit.

### Statistical analysis

Continuous data are presented as the median with interquartile range (IQR), while categorical data are expressed as percentages.

All statistical analyses were conducted using SPSS software, version 23.0 (IBM, Armonk, NY, USA).

## Results

### Characteristics of respondents

A total of 424 out of 2560 endocrinologists participated in the survey, resulting in a response rate of 16.6%. The characteristics of the participants in the 2024 survey and those who took part in the 2022 survey are reported in **Table 1**. Almost two-thirds of participants were females. The sample was well-balanced in terms of age groups and years of practice. The primary areas of practice included thyroid disorders, general endocrinology and diabetes mellitus. Additionally, 32.5% of respondents identified nutrition and obesity as a key area of interest.

**Table 1:** Characteristics of endocrinologists participating to the 2022 and 2024 surveys.

Characteristics	2022	2024
	N (%)	N (%)
Gender		
Male	218 (40.2%)	164 (38.7%)
Female	318 (58.7%)	256 (60.4%)
Not reported	6 (1.1%)	4 (0.9%)
Age (years)		
<30	115 (21.5%)	27 (6.4%)
30-40	97 (18.2%)	102 (24.1%)
41-50	135 (25.3%)	72 (17.0%)
51-60	125 (23.4%)	82 (19.3%)
61-70	18 (3.4%)	95 (22.4%)
>70	37 (6.9%)	45 (10.6%)
Not reported	7 (1.3%)	1 (0.2%)
Years of practice in the endocrinologic/metabolic field		
<5	65 (12.2%)	61 (14.4%)
5-10	72 (13.5%)	77 (18.2%)
11-20	121 (22.7%)	79 (18.6%)
21-30	110 (20.6%)	75 (17.7%)
31-40	111 (20.8%)	83 (19.6%)
>40	35 (6.6%)	48 (11.3%)
Not reported	20 (3.7%)	1 (0.2%)
Main areas of practice*		
Thyroid	349 (65.4%)	267 (63.0%)
General endocrinology	284 (53.2%)	231 (54.5%)
Diabetes mellitus	253 (47.4%)	234 (55.2%)
Nutrition/obesity	157 (29.4%)	138 (32.5%)
Osteoporosis	90 (16.9%)	88 (20.8%)
Pituitary gland	51 (9.6%)	32 (7.5%)
Andrology	24 (4.5%)	26 (6.1%)
Adrenal gland	39 (7.3%)	23 (5.4%)
Mineral metabolism	29 (5.4%)	22 (5.2%)
Oncological endocrinology	42 (7.9%)	19 (4.5%)
Pediatric endocrinology	16 (3.0%)	13 (3.1%)
Reproduction	12 (2.2%)	9 (2.1%)
Endocrinological surgery	4 (0.7%)	1 (0.2%)
Kind of practice		
Private practice	164 (30.7%)	148 (34.9%)
Hospital	206 (38.6%)	132 (31.1%)
Outpatient clinic	79 (14.8%)	81 (19.1%)
Resident doctor	30 (5.6%)	37 (8.7%)
University	16 (3.0%)	11 (2.6%)
Setting of practice		
Non-university hospital	188 (35.2%)	131 (30.9%)
University hospital	96 (18.0%)	72 (17.0%)
Public outpatient clinic	89 (16.7%)	81 (19.1%)
Private outpatient clinic	129 (24.2%)	129 (30.4%)
Other	32 (6.0%)	2 (0.5%)
Availability in the structure of a specialist or unit dedicated to obesity		
Yes	278 (52.1%)	209 (49.3%)
No	210 (39.3%)	194 (45.8%)
Don’t know	13 (2.4%)	12 (2.8%)
Not reported	33 (6.2%)	9 (2.1%)

NR, not reported.

*more than one answer was allowed.

Almost half of the surveyed endocrinologists worked in a public hospital setting, with 17.0% affiliated with a university and 30.9% in non-affiliated hospitals. The remaining participants practiced in public (19.1%) or private (30.4%) outpatient clinics. A dedicated obesity specialist or unit was present in the practice setting of 49.3% of the participants.

Compared to the participants in the 2022 survey, the sample involved in the new survey showed a lower prevalence of endocrinologists under the age of 30 and a higher prevalence of specialists who were older and had longer work experience in the endocrine-metabolic field.

### Management of obesity

Responses to this section of the survey in comparison with those of the 2022 survey are summarized in **Table 2**. Among participants, 39.6% reported that 20% to 35% of the patients they see each month have obesity, while 43.4% indicated even higher percentages, compared to 37.8% in the previous survey.

**Table 2:** Comparison of attitudes and practices in obesity: 2022 vs. 2024 surveys.

Item	2022 survey	2024 survey
How many patients, among those you see in a month, have obesity?		
<20%	17.5%	14.4%
20-35%	44.7%	39.6%
36-50%	24.9%	31.1%
>50%	12.9%	12.3%
During the visit of a patient with obesity who came for another endocrinological problem, how often is the problem of obesity addressed?		
Never	1.8%	2.8%
In 25% of the cases	20.7%	18.9%
In 50% of the cases	22.5%	21.5%
Il 75% of the cases	18.5%	18.4%
Always	36.5%	35.4%
How do you manage obesity in a patient who arrived at your clinic for diseases other than obesity?		
I manage it personally	29.1%	30.4%
I manage it in the context of a dedicated team	13.5%	15.1%
I refer the patient to a dietician/nutritionist in the center where I work	29.7%	26.9%
I refer the patient to a dietician/nutritionist in another center	15.0%	13.7%
I refer the patient to a 2^nd^ level center for obesity therapy	6.6%	6.8%
I invite the General Practitioner to deal with the problem	1.4%	0.9%
Other	4.7%	2.4%
Who refers patients with obesity to you? *		
General Practitioners	54.1%	52.1%
Other specialists	31.0%	31.6%
Direct access	41.5%	39.2%
Nobody refers to me this kind of patients	16.9%	13.4%
When do you think it is useful to prescribe a ketogenic diet?		
Never	15.2%	12.0%
Always	5.0%	4.7%
As a first approach after the failure of lifestyle intervention	41.8%	39.2%
In case of failure of pharmacological treatment	15.8%	9.7%
As bridge therapy before bariatric surgery	22.3%	28.5%
What is the usefulness of substitute foods? *		
None	10.0%	6.6%
Complementary	19.7%	24.5%
The cost limits their use	35.8%	33.7%
It depends on the individual patient	37.7%	31.6%
I do not know	12.1%	10.1%
What type of exercise do you recommend to your patient with obesity? *		
Anaerobic exercises	3.8%	4.2%
Aerobic exercises	45.9%	37.0%
Strength exercises	1.8%	3.5%
Flexibility exercises	1.5%	0.7%
Individualized exercises in relation to the type of patient	47.7%	43.6%
The duration and intensity of the exercise are important, not the type	11.3%	7.1%
Mixed program that includes all types of exercise	17.7%	21.7%
Exercise rarely solves the problem	2.9%	3.3%
Who do you think should be recommended psychological support in the management of obesity?		
Everyone	41.9%	38.0%
Over half of the cases	32.2%	30.2%
25-50% of the cases	16%	14.2%
< 25% of the cases	6.9%	5.7%
Only in the presence of psychiatric problems	3.0%	2.1%

*More than one answer allowed.

No major differences between the two surveys emerged regarding the proportion of patients with obesity seeking care for another endocrinological issue in whom obesity was addressed. On the other hand, the proportion of endocrinologists who reported managing obesity themselves, either independently or as part of a dedicated team, slightly increased from 42.6% to 45.5%.

Beliefs regarding the role of ketogenic diet and physical activity did not change with respect to the 2022 survey. In particular, a large majority of respondents found the ketogenic diet useful, particularly as an initial approach following the failure of lifestyle interventions (39.2%), or as bridge therapy before bariatric surgery (28.5%).

The most frequently recommended types of exercise included individualized programs tailored to the patient’s needs (43.6%) and aerobic exercises (37.0%).

Psychological support was viewed as a valuable component in obesity management. Among participants, 38.0% believed it should be offered to all patients with obesity, while an additional 30.2% recommended it in at least half of the cases. No major changes were evidenced with respect to the previous survey.

### Pharmacological treatment of obesity

**Table 3** summarizes attitudes and practices relative to the treatment of obesity.

**Table 3:** Comparison of attitudes and practices in obesity pharmacological treatment: 2022 vs. 2024 surveys.

Item	2022 survey	2024 survey
What is your first approach to treating patients with BMI between 30 and 34 kg/m^2^?		
Pharmacological	65.2%	75.7%
Surgical (only with comorbidities)	9.8%	4.7%
Referral to a 2^nd^ level center	–	1.2%
What is your first approach to treating patients with BMI between 35 and 39 kg/m^2^?		
Pharmacological	38.5%	49.8%
Surgical	18.0%	3.8%
Surgical (only with comorbidities)	5.1%	11.3%
Referral to a 2^nd^ level center	32.6%	26.7%
What is your first approach to treating patients with BMI ≥40 kg/m^2^?		
Pharmacological	12.6%	20.0%
Surgical	16.5%	24.8%
Surgical (only with comorbidities)	26.1%	11.1%
Referral to a 2^nd^ level center	43.3%	37.5%
In what percentage of patients do you use:		
Metformin	30 [10-50]	30 [10-50]
Orlistat	0 [0-10]	0 [0-5]
Naltrexone/Bupropion	0 [0-5]	0 [0-5]
Liraglutide	10 [0-30]	25 [5-50]
What is the major obstacle to prescribing drug therapy for obesity?		
Side effects	34.6%	20.0%
Cost	71.9%	80.0%
Resistance on the part of the patient	21.9%	14.2%
Poor effectiveness	14.5%	4.7%
Limited durability	25.6%	20.3%
Distrust on the prescriber	6.5%	4.5%
What factors negatively affect adherence and therefore the persistence of the results of drug therapy?*		
Side effects	44.9%	35.6%
Cost	64.9%	72.4%
Insufficient motivation	55.3%	38.9%
Therapeutic failure	38.1%	26.7%
Distrust of the prescriber	6.5%	5.4%
In what percentage of cases do you think obesity can be successfully managed in the long term with lifestyle intervention combined with drug therapy?		
<10%	13.4%	8.8%
10-30%	38.0%	27.8%
31-50%	28.5%	27.1%
>50%	20.1%	27.4%
Are you aware of new drugs coming for obesity therapy?		
Yes	40.5%	77.8%
Do you think the availability of new drugs:		
Will increase the number of patients candidate to drug therapy	56.1%	55.2%
Will improve the effectiveness of drug therapy	36.6%	50.0%
Will improve the durability of drug therapy	30.4%	28.1%
Will reduce the costs of treatment	12.6%	13.9%
Will not lead to substantial changes	7.6%	5.2%

In the 2024 survey, the first approach to treating patients with BMI between 30 and 34 kg/m^2^ was pharmacological treatment (associated with lifestyle interventions) for 75.7% of participants, as compared to 65.2% in the previous survey. Also, for patients with BMI between 35 and 39 kg/m^2^ the proportion of respondents preferring pharmacological treatment as the first option increased from 38.5% in 2022 to 49.8% in 2024. In patient with severe obesity (BMI ≥40 kg/m^2^), surgery or referral to a specialized center remained the commonest approaches. However, even in this case the choice of pharmacological treatment as initial approach rose from 12.6% to 20.0%.

Regarding the type of pharmacological treatment, the reported use of metformin, orlistat, and naltrexone/bupropion remained unchanged. However, there was a significant increase in the use of liraglutide. Specifically, its average usage rose from 10% in 2022 to 25% in 2024. Moreover, the upper quartile reached 50%, indicating that one-fourth of participants prescribed liraglutide to at least half of their patients with obesity. The perception of the obstacles to the prescription of drug therapy for obesity also changed; in fact, the proportion of respondents reporting side effects, resistance on the part of the patient, poor effectiveness or lack of durability decreased, while the percentage of those considering cost of treatment as the major barrier increased from 71.9% to 80.0%. Expectations for successfully managing obesity in over 50% of patients through a combination of lifestyle interventions and drug therapy increased from 20.1% to 27.4%. Additionally, the percentage of endocrinologists aware of upcoming obesity treatments significantly rose from 40.5% in 2022 to 77.8% in 2024. Expectations for enhanced effectiveness with new medications also rose from 36.6% to 50.0%.

## Discussion

The findings of this extensive survey highlight the significant impact of obesity on the clinical practice of Italian endocrinologists. Over 40% of respondents indicated that one in three to over half of patients attending their wards have obesity, with an increasing trend in comparison with the previous survey. However, one in five endocrinologists still address obesity in only 25% or fewer of the patients seeking care for other endocrinological conditions.

Our survey documented changes in endocrinologists’ attitudes and practices regarding the treatment of obesity. In particular, the proportion of participants considering pharmacological treatment as the initial approach to patients with obesity increased, while fewer prioritized surgery or referral to specialized centers as the primary strategy. This suggests that the availability of safe and effective anti-obesity drugs, along with the educational activities promoted by AME, may have encouraged endocrinologists to more frequently consider pharmacological treatment as an initial approach, along with lifestyle interventions.

The percentage of endocrinologists referring patients to specialized centers increased with BMI. However, even in cases of severe obesity, only one-third of participants considered bariatric surgery as the primary treatment option. These findings emphasize the need for a structured, multidisciplinary approach to obesity management, integrating shared diagnostic and therapeutic pathways for comprehensive patient care.

The survey underscores the significant underutilization of anti-obesity medications in routine clinical practice, despite a promising increase in liraglutide use compared to the previous survey. These findings reflect the increasing body of evidence supporting the beneficial effects of GLP1 receptor agonists not only for weight reduction, but also for cardio-renal protection (25).

The overall picture emerging from this survey, conducted two years apart from the previous one, suggests a greater awareness of endocrinologists about the potential of treating patients with obesity with GLP1 receptor agonists, with less worries about side effects and patient resistance. Of note, poor effectiveness almost disappeared among the reported obstacles to prescribing anti-obesity drugs. The different distribution of responders, characterized by greater field experience, may have at least partially influenced the higher prescribing attitude toward liraglutide observed in the survey. However, the percentage increase in liraglutide prescribers in this survey is much greater than the increase in participants with long-standing experience. Also, compared with the previous survey, the proportion of participants aware of upcoming new anti-obesity medications increased substantially from 40.5% to 77.8%, suggesting a growing interest of endocrinologists for the management of obesity. Furthermore, respondents manifested greater expectations regarding the possibility of achieving long-term success through a combination of lifestyle interventions and new drug therapies.

However, these changes in attitudes only partially translated into changes in behavior. In fact, compared to the previous survey, there was no clear increase in the tendency to take responsibility for the management of people with obesity, and the use of medications remained limited. It is plausible that translating knowledge into practice requires more time and more intensive educational interventions. Furthermore, perceived barriers to drug prescription still persist. In this respect, the cost of treatment can limit a wider use of new anti-obesity drugs and was identified as a major obstacle by 80.0% of participants, significantly affecting adherence to long-term therapy. Notably, the lack of reimbursement by the Italian National Health System further complicates access to sustained treatment, presenting a significant challenge for patients. Other commonly cited barriers to drug therapy included concerns about side effects and the limited durability or effectiveness of treatment. The perception that existing weight-loss medications may not be safe or effective aligns with previous findings (17, 26) and may partly explain the low prescription rates. However, comparison with the previous survey suggests that confidence in new treatments is increasing. Additionally, 14% of participants identified patient resistance as an obstacle to prescribing anti-obesity drugs, while 38.9% of the respondents cited insufficient patient motivation as a key reason for poor adherence and long-term treatment outcomes. Several factors may contribute to patients’ reluctance to pursue pharmacological treatment, including high costs, side effects, and perceived limited efficacy. Moreover, a lack of recognition of obesity as a chronic condition, difficulties in viewing weight-loss treatment as a long-term commitment, social and environmental challenges that hinder weight loss, and the presence of multiple comorbidities can all contribute to patient resistance (27). Therefore, endocrinologists should be trained to recognize treatment barriers early to improve the likelihood of long-term success.

Our study has some limitations. First, the response rate was 16.6%, which may reflect limited awareness about obesity management. However, previous surveys have reported response rates ranging from 10.8% to 34% (19, 28–31). Additionally, the survey sample may not fully represent the broader community of endocrinologists. However, the respondents’ sex, age, regional distribution, and professional experience were consistent with the overall demographics of AME members. Two new and very effective anti-obesity drugs, semaglutide and tirzepatide, are presently marketed in Italy but were not available when the survey was conducted. Lastly, participants in the survey may have been those with a greater interest in obesity management, potentially leading to an optimistic picture of obesity-related attitudes and practices among Italian endocrinologists.

In conclusion, our survey highlights that while endocrinologists frequently encounter patients with obesity, they often do not actively address or manage the condition. Since individuals with obesity may not recognize the need for weight reduction until it affects their health, it is crucial for endocrinologists to initiate conversations about weight early on to facilitate timely interventions and prevent obesity-related complications.

Although currently available anti-obesity medications are safe and effective, with even more promising treatments on the horizon, pharmacological therapy remains underutilized. However, there are indications of a growing adoption of new therapies in clinical practice. In this respect, a dedicated educational program should strengthen endocrinologists’ familiarity with new drugs and promote better recognition of bariatric surgery indications. Addressing the unmet educational needs of endocrinologists is essential to enhance their awareness of obesity and increase their confidence in managing this widespread condition, which has a profound impact on society.

## Data Availability

The raw data supporting the conclusions of this article will be made available by the authors, without undue reservation.
